# Natural Biomolecules and Light: Antimicrobial Photodynamic Strategies in the Fight Against Antibiotic Resistance

**DOI:** 10.3390/ijms26167993

**Published:** 2025-08-19

**Authors:** Greta Amendola, Mariagrazia Di Luca, Antonella Sgarbossa

**Affiliations:** 1 National Research Council-Nanoscience Institute (CNR-NANO) and NEST-Scuola Normale Superiore, Piazza S. Silvestro 12, 56127 Pisa, Italy; greta.amendola@nano.cnr.it; 2 Department of Biology, University of Pisa, 56127 Pisa, Italy; mariagrazia.diluca@unipi.it

**Keywords:** antimicrobial resistance, antimicrobial photodynamic therapy, natural photosensitizers

## Abstract

The alarming increase in infections caused by antimicrobial-resistant bacteria is increasingly posing a critical threat to public health. For this reason, the scientific community is focusing on alternative therapeutic strategies, such as antimicrobial photodynamic therapy (aPDT). This review examined the use of natural photosensitizers (PSs) in aPDT, emphasizing how they may produce high yields of reactive oxygen species when activated by light and consequently inactivate a wide range of pathogens, including bacteria embedded in biofilms, efficiently. The main methodologies and several strategies of incorporation into cutting-edge nanotechnological delivery systems of the most prevalent natural PSs (curcuminoids, perylenequinones, tetrapyrrolic macrocycles, and flavins) have been analyzed. Although natural PSs have benefits in terms of environmental sustainability and biocompatibility, their clinical use is frequently constrained by low bioavailability and solubility, issues that are being addressed more and more through novel formulations and dual-mode treatments. Studies conducted both in vitro and in vivo highlight these compounds’ strong antibacterial and wound-healing properties. In conclusion, natural molecule-based aPDT is a flexible and successful strategy for combating antimicrobial resistance, deserving of more translational study and clinical advancement.

## 1. Introduction

Since the discovery of penicillin in 1928 by Alexander Fleming and the following ‘golden era’, antibiotics have represented the principal antimicrobial agents for treating and controlling infectious diseases [1]. In the subsequent decades, the widespread and often inappropriate use of antibiotics has significantly contributed to the emergence of antimicrobial resistance (AMR) [2,3]. The main causes are the unregulated sales of antibiotics, inadequate sanitation practices, and uncontrolled environmental discharge, including agricultural usage, animal healthcare, and food systems. Nowadays, the World Health Organization (WHO) recognizes AMR as one of the most serious global health threats of the 21st century. In 2019 alone, an estimated 4.95 million deaths worldwide were associated with bacterial AMR, and projections suggest this number could rise to 10 million deaths annually by 2050 [4]. AMR can occur in bacteria, viruses, fungi, and parasites. In particular, bacteria may acquire antibiotic resistance by co-existence, genomic mutations after selection pressure, or horizontal transfer of a large range of antibiotic resistance genes [5]. The primary mechanisms of AMR include the production of enzymes that inactivate antibiotics, limited drug uptake due to altered porin expression, activation of efflux pump systems, decreased membrane permeability, and modifications in metabolic pathways and molecular targets [6]. Moreover, the formation of complex bacterial communities known as biofilms significantly contributes to both antibiotic resistance and tolerance. Within these structures, bacterial cells—either of a single species or multiple species—adhere to biotic or abiotic surfaces and become embedded in a highly hydrated extracellular matrix primarily composed of extracellular polymeric substances (EPS) [7]. Firstly, the biofilm matrix offers protection against a variety of external and internal environmental stresses, including host immune responses, antimicrobial agents, and mechanical forces [8]. Furthermore, nutrient depletion and the accumulation of waste products in the biofilm environment lead to reduced bacterial growth rates and the formation of “persister cells”. This metabolic downshift diminishes the activity of key cellular processes such as DNA replication, cell division, and protein synthesis, thereby impairing the efficacy of antibiotics targeting these functions [9]. Finally, bacteria within biofilms may upregulate the expression of antimicrobial resistance genes, leading to the overproduction of specific proteins or biopolymers that further enhance their defensive capabilities [10].

Given the significant impact of AMR on public health and the limited and delayed development of new antibiotics due to the pressure of cost and market productivity, the scientific community is focusing on alternative antimicrobial strategies. Antimicrobial photodynamic therapy is a promising approach to eradicate difficult-to-treat bacterial infections thanks to its broad antimicrobial spectrum, low toxicity, and minimal side effects [11]. This strategy exploits the property of single molecules or complexes, known as photosensitizers, to absorb visible light and induce lethal oxidative stress, which causes cell death. PSs could be natural or synthetic compounds, and the choice between them is determined by costs, availability, and therapeutic goals. Nowadays, scientific research is investigating the application and optimization of natural molecules due to their environmental advantages, biocompatibility, and lower toxicity. This review aims to give an overview of the application of natural photosensitizers in aPDT to treat different antibiotic-resistant bacteria.

## 2. Antimicrobial Photodynamic Therapy

Since ancient Egypt and Greece, photodynamic therapy has been used to treat some skin diseases, such as leprosy lesions, by applying natural extracts photoactivated with sunlight. Photodynamic therapy was introduced in medical practices in 1900 thanks to Oscar Raab, who observed the toxic action of the dye acridine on Paramecium species after irradiation with daylight [12].

**Figure 1:**
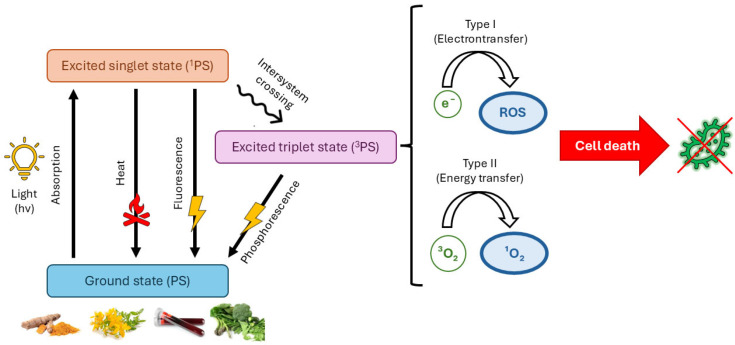
Simplified Jablonski diagram representing the essential photochemical and photophysical processes of antimicrobial photodynamic treatment (aPDT).

In aPDT, there are three main protagonists: photosensitizers, light source, and molecular oxygen. Photosensitizers are natural or artificial molecules capable of absorbing light within the spectral range from visible to near-infrared wavelengths. Once PS at its ground state is irradiated by a proper wavelength, it can absorb a photon and promote to the electronically excited singlet state (^1^PS). The PS in its singlet excited state is unstable and can release energy through fluorescence emission or production of heat, lapsing to its ground state. However, for the photodynamic effect, the spin inversion occurring within the excited state, called “intersystem crossing”, is the most relevant process. Consequently, a more stable excited triplet state is formed, and the longer lifetime of this state allows for the transfer of energy or electrons to very close molecular oxygen. The following photochemical reactions proceed via type I or type II processes. In the type I mechanism, the formation of reactive oxygen species (ROS), including hydroxyl radicals, superoxide anions, and hydrogen peroxide, is initiated by the electron transfer from the triplet state PS to molecular oxygen, giving rise to a series of oxidative stresses, causing cell damage and ultimately death. Whereas the transfer of energy to molecular oxygen in its triplet state results in the formation of the highly reactive singlet oxygen (^1^O_2_), giving birth to the type II photochemical reaction. The relative occurrence of these two types depends on the specific photosensitizer employed and on the environmental conditions under which aPDT is performed [13,14] (Figure 1).

APDT offers several advantages over conventional antibiotics. First, it has a broad antimicrobial spectrum, targeting various metabolic pathways and resulting in success against bacteria, fungi, and protozoa. Then, a major benefit is its triple site-specificity, which arises from: (1) the preferential uptake or accumulation of PSs by target cells over non-target cells; (2) the pharmacodynamic inactivity of non-irradiated PSs; and (3) the spatial confinement of light exposure to the infected area. As a result, systemic toxicity is largely avoided outside the irradiated, PS-rich region. Despite antimicrobial photodynamic treatment (aPDT) being seen as a viable alternative to traditional antibiotics due to its minimal risk of resistance development, current research has shown many bacterial adaptive responses that may facilitate tolerance. These include the production of biofilms that physically protect bacterial cells from light and ROS, the activation of efflux pumps that drive out photosensitizers, and the modification of quorum-sensing pathways that control reactions to oxidative stress. ROS may also be neutralized by antioxidant defense systems, as well as by elevated catalase and superoxide dismutase expression. Repeated exposure to sublethal aPDT dosages has been linked to genetic changes and changed phenotypes that imply the possibility of adaptive tolerance under selection pressure, even if these mechanisms do not yet represent conclusive resistance [15].

## 3. Natural Photosensitizers

**Figure 2:**
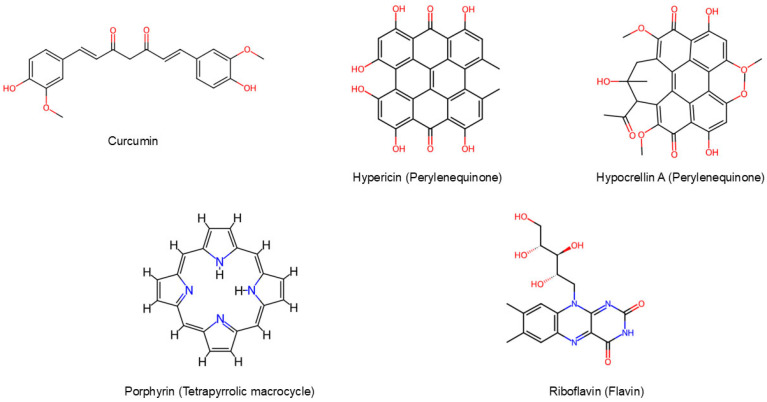
Molecular structures of natural PSs. Figures downloaded from ChemSpider.

Natural photosensitizers represent safe and sustainable molecules thanks to their biocompatibility, low toxicity, environmental sustainability, and natural abundance. Moreover, they are preferred to synthetic ones because they usually do not need prolonged treatments, avoiding the generation of toxic waste [16]. Plants, animals, algae, fungi, protozoa, and bacteria are sources of many natural photoactive molecules. Curcuminoids, perylenequinones, tetrapyrrolic macrocycles, and flavins are the most commonly employed in photodynamic therapy against Gram-positive and Gram-negative bacteria [17] (Figure 2). However, the limited solubility of natural photosensitizers presents a significant challenge to the broader implementation of aPDT. The hydrophobic nature of these compounds negatively affects their bioavailability and hinders their efficient uptake by cells. Additionally, inadequate solubility increases the susceptibility of PSs to hydrolytic degradation and promotes aggregation before reaching target cells. This aggregation leads to fluorescence quenching, ultimately reducing the generation of reactive oxygen species (ROS). As a result, the overall therapeutic efficacy of aPDT is diminished. These issues highlight the urgent need for effective strategies to enhance the solubility of PSs [16]. In Table 1 the main properties of the natural photosensitizers discussed in this review have been summarized.

### 3.1. Curcuminoids

Curcuminoids, such as curcumin (CUR), bisdemethoxycurcumin, and dimethoxycurcumin, are bioactive polyphenols extracted from the rhizomes of Curcuma longa, a member of the Zingiberaceae family commonly known as turmeric. This plant is widely distributed in tropical areas, particularly across South Asia and the Indian subcontinent [20]. Curcumin is well known for its biomedical applications as an antioxidant, anti-inflammatory, antimutagenic, anticarcinogenic, antithrombotic, antiviral, and antimicrobial [21]. Among these properties, curcumin represents a highly effective PS photoactivated by blue light, exhibiting a wide absorption spectrum within the 300–500 nm wavelength range with a maximum at 430 nm [22]. Thanks to these characteristics and their nontoxicity in cell culture models [23] and animal studies, curcumin and curcuminoids have been widely explored as PS for aPDT, both against Gram-positive and Gram-negative bacteria. The antibacterial activity might be related to the alteration of the functionality of membrane proteins, the modification in gene expression, the inhibition of DNA replication, and the reduction of motility [20,24].

Curcumin-mediated photodynamic therapy exhibited considerable antibacterial activity against numerous Gram-positive bacteria, such as *Staphylococcus aureus* (including methicillin-resistant (MRSA) and vancomycin-resistant (VRSA) strains), *Enterococcus faecalis*, *Streptococcus mutans*, and *Propionibacterium acne*. By means of scanning electron microscopy studies and fluorescence assays, it has been shown that curcumin photoactivated by blue LED light (450–460 nm) at energy fluences between 10 and 180 J/cm^2^ was able to produce elevated levels of ROS, particularly singlet oxygen, which damaged bacterial membranes and intracellular structures. In particular, curcumin-based aPDT could be exploited in the food industry for killing foodborne pathogens. In fact, substantial reductions in viable *S. aureus* populations, with log decreases of up to 6.9, were observed in both buffer solutions and various food matrices [24,25,26,27,28,29,30]. Furthermore, photoactivated curcumin was able to inhibit biofilm formation in resistant *S. aureus* populations, downregulating virulence-associated gene expression and increasing antibiotic sensitivity [31,32]. For instance, curcumin concentrations of 1.5–3 mg/mL and light dosages of 72 J/cm^2^ have been shown to reduce *S. mutans* and *E. faecalis* colony-forming unit (CFU) counts by over 90% in both planktonic and biofilm stages [30,33].

Gram-negative bacteria generally exhibit lower sensitivity to curcumin compared to Gram-positive bacteria, due to the presence of an outer membrane composed of lipopolysaccharides, which acts as a barrier to the penetration of the dye. Combining curcumin with a membrane permeability enhancer might be an intriguing strategy for overcoming this challenge. In particular, natural borneol, a natural and safe molecule used as an adjuvant to improve the efficiency of drug absorption, in association with 12.5 μM of curcumin activated by 425 nm LED light at 6.5 J/cm^2^, resulted in ROS-mediated cell membrane rupture and a decrease of up to 4.41 log_10_ CFU/mL in *Escherichia coli* [34]. Similarly, curcumin alone at 6.75 mM irradiated with 10 J/cm^2^ of 405 nm light dramatically decreased the thickness of biofilms in *Pseudomonas aeruginosa*, interfered with the expression of genes involved in quorum sensing, and prevented the synthesis of extracellular polysaccharides [35].

3,3′-dihydroxycurcumin (DHC), a more stable and less colored curcumin analog, exhibited superior antimicrobial activity, particularly evident with photoactivation of the molecules using LED light (440–480 nm, 100 mW/cm^2^, 60 s) on *S. mutans*, *Lactobacillus casei*, *Actinomyces israelii*, *Fusobacterium nucleatum*, and *E. faecalis* cultures. However, both DHC and curcumin significantly reduced the bacterial growth of initial (72 h) and mature (7 days) single biofilms, with the effect enhanced by LED light, particularly in the case of *F. nucleatum* and *E. faecalis*. The best antimicrobial activity of irradiated DHC was observed on biofilm inside dentinal tubules, reducing 76.5% of the microorganisms. Instead, for the biofilm on necrotic root canals, DHC and CUR were able to reduce 45.8% and 48.24% of dead cells when irradiated, respectively. As a bioactive molecule, DHC, due to its minimal cytotoxicity and antibiofilm action comparable to curcumin, may be further investigated for endodontic applications [36].

#### 3.1.1. Curcumin in Combination with Nanostructures

**Figure 3:**
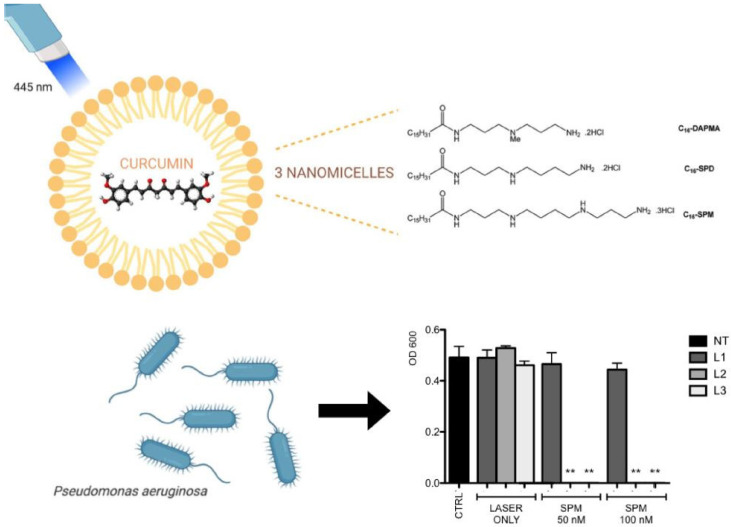
Scheme of the structure of nanomicelles loaded with curcumin and antimicrobial photodynamic therapy against *P. aeruginosa* irradiated with blue laser light at different dosages (NT, L1, L2, L3). In the graph, ** represent Mann–Whitney U test *p* < 0.001 (treatment vs control). Figure reprinted from [38] under Creative Commons License. Copyright © 2020 Wiley-VCH GmbH.

Although photoactivated curcumin has an effective antimicrobial activity, it has some limitations for clinical applicability, mostly connected to its hydrophobicity, low absorption, fast metabolism, and chemical instability [36]. To overcome these limitations, scientists are focusing on different drug delivery systems. With the increasing development of nanotechnology, many strategies and methodologies have been applied to increase curcumin’s solubility and bioavailability [37]. One of these is using amphiphilic nanostructures, which allow the encapsulation of hydrophobic curcumin and make it more soluble in water with their external hydrophilic portion. Rupel et al. demonstrated that curcumin encapsulated with three different amphiphilic molecules with C16-long hydrocarbon chain and N,N-di-(3-aminopropyl)-N-methylamine (DAPMA), spermidine (SPD), and spermine (SPM) as hydrophilic portions was able to inhibit *P. aeruginosa* at 500 nM, 1 µM and 250 nM, respectively, compared to the effect of CUR alone, which can reduce the bacterial growth of approximately 50% at the concentration of 50 µM. Moreover, the activation of curcumin with blue laser (0.1 W/cm^2^ for 180–300 s) did not affect the bacterial growth; on the other hand, the irradiation of CUR-SPM nanomicelles completely inhibited the bacterial growth even at the lowest concentration (50 nm). It is plausible to suppose that curcumin’s interaction with *P. aeruginosa*’s outer membrane is improved when inserted in nanomicelles. As for the biocompatibility, all CUR-loaded nanosystems were found to be completely nontoxic on human keratinocytes, even at the maximum tested concentration (1 µM), unlike free curcumin, which negatively affected cell viability from 20 µM (Figure 3) [38].

Similar results were obtained on *E. faecalis*, *Actinomyces viscosus*, and *Streptococcus oralis* by encapsulating curcumin in polymeric nanoparticles (NP) of poly (lactic-co-glycolic acid) (PLGA) (average size of 248 nm and encapsulation efficiency of 98.07%). The NP-CUR photoactivated with blue LED (22 mW/cm^2^, 450 nm excitation wavelength, 55 min) reduced the minimum inhibitory concentration (MIC) and minimum bactericidal concentration (MBC) in all tested strains compared to free curcumin, and the same effect was also observed in the treatment of single- and multispecies biofilms. Additionally, curcumin at the concentration of 500 µg/mL showed a cytotoxic effect, reducing the cell viability to less than 20%; whereas, the NP-CUR treatment proved an optimal biocompatibility and a putative stimulating effect on cell proliferation (more than 100% cell viability) [22].

In other studies, the antimicrobial photodynamic therapy with curcumin nanostructures was enhanced with photothermal therapy (PTT), demonstrating a significantly improved antibacterial efficacy, particularly against bacteria resistant to conventional antibiotics. Nanostructures and plasmonic metals that exhibit strong absorption in the visible and near-infrared spectrum and have the capacity to convert absorbed light into heat are known as photothermal antimicrobial agents. Heating microbial pathogens over 45 °C reduces the viability of most bacteria [39]. It was shown that curcumin, when integrated into engineered nanoplatforms such as chitosan-coated gold nanoparticles (AuNPs/CS-Cur), nitrogen-doped carbon dot composites (CDs-Cur), or hybrid hydrogels (AC-Gel@Cur-Au), better combats bacterial infections under dual light irradiation (405 nm and 808 nm). These nanostructures were able to produce substantial reactive oxygen species (ROS) and heat, leading to effective disruption of bacterial membranes. In particular, up to 99.99% killing effectiveness against *S. aureus* and *E. coli* was made possible by the positively charged chitosan coating in the AuNPs/CS-Cur system, which enhanced curcumin stability and promoted electrostatic attachment to bacterial surfaces [40]. Moreover, CDs-Cur at nanomolar quantities (1 nM) eliminated both *S. aureus* and *E. coli*, significantly outperforming free curcumin [41]. Similarly, hydrogels loaded with curcumin demonstrated strong, stimuli-responsive antibacterial activity, which included effective destruction of biofilms and faster wound healing in vivo [42]. These results highlight the promise of dual-mode phototherapeutic systems based on curcumin as effective, safe, and biocompatible substitutes for traditional antimicrobial treatments. In addition, nanocarriers improve the pharmacological profile of curcumin and make photodynamic antibacterial treatment more effective, targeted, and long-lasting against a variety of pathogens. These nanosystems show strong potential for future clinical translation, especially against biofilm-related infections, chronic wounds, and drug-resistant microbes [43].

#### 3.1.2. In Vivo Studies

Curcumin has been widely studied in vitro for its minimal toxicity on non-transformed cell lines. These findings show that normal cells exhibit minimal cytotoxic reactions even when exposed to high doses of curcumin over lengthy periods of time. The pharmacokinetic behavior of curcumin is characterized by rapid metabolism and poor absorption, particularly when administered orally [44]. Animal research supports these findings. For example, toxicity testing in rats and mice, including acute and sub-chronic investigations, has shown that high-dose curcumin formulations are well tolerated. One study found that after administering up to 5 g/kg body weight in animals, no substantial toxicity was observed [45].

The use of curcumin-based nanostructures in antimicrobial photodynamic therapy (aPDT) has shown significant in vivo efficacy in a variety of preclinical models of bacterial infection, providing a powerful non-antibiotic approach to the treatment of multidrug-resistant and biofilm-associated infections. Ghorbanpour et al. created curcumin-loaded poly (lactic-co-glycolic acid) nanoparticles (Cur-PLGA-NPs) and activated them with blue LED light (450 ± 5 nm) to target *S. mutans* colonization in a rat model of orthodontic appliance-associated caries. Over 30 days, aPDT with Cur-PLGA-NPs dramatically decreased the bacterial load and inhibited the expression of the *gtfB* gene, which encodes for glucosyltransferase-I and is responsible for the formation of dental caries [46].

**Figure 4:**
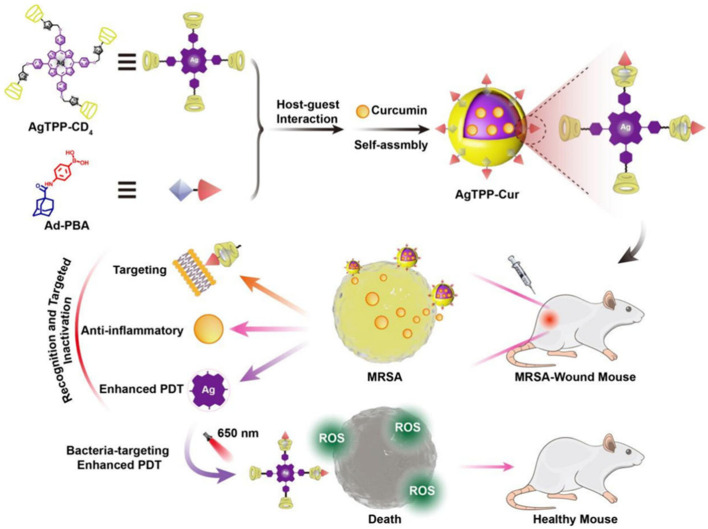
Schematic representation of AgTPP-Cur formation and antimicrobial photodynamic application on a mouse wound model infected with MRSA. Reprinted with permission from [47]. Copyright © 2025 American Chemical Society.

The combination of curcumin with silver-porphyrin cyclodextrin and phenylboronic acid created a supramolecular nanophotosensitizer (AgTPP-Cur) that targets MRSA. This technology produced a lot of reactive oxygen species (ROS), broke down MRSA membranes, and decreased the number of microorganisms in infected wounds when activated by near-infrared (650 nm) light. In vivo, mice treated with AgTPP-Cur aPDT exhibited a 93% wound closure rate after 9 days, with no systemic toxicity and little inflammation. Crucially, repeated sublethal exposures of AgTPP-Cur did not result in antimicrobial resistance, highlighting its safety and viability as a treatment option (Figure 4) [47].

In a separate in vivo burn wound model, a dual-function nanoplatform including curcumin and nisin in a poly (L-lactic acid) nanoparticle (CurNisNP) was administered via combined light and ultrasonic exposure (photo-sonodynamic treatment, or aPSDT). The formulation showed no cytotoxicity to normal fibroblasts and prolonged drug release. In comparison to silver sulfadiazine, a common topical antibiotic, CurNisNP-mediated aPDT significantly downregulated virulence genes (*abaI*, *csuE*), upregulated the *blsA* photoreceptor gene, and improved wound healing and re-epithelialization when applied to *Acinetobacter baumannii*-infected wounds [48].

Similarly, Faghani-Eskandarkolaei et al. developed a gold-curcumin-polydopamine nanohybrid (GCDNH) as a dual photo-sonosensitizer in order to eradicate *S. aureus* from contaminated implants. In vitro tests using GCDNH showed 98% antibacterial and 99% anti-biofilm efficiency when subjected to both ultrasonic and 808 nm near-infrared light. The GCDNH aPSDT treatment effectively eradicated bacterial colonization in a rat subcutaneous implant model without causing harm to the surrounding tissue. The approach’s tissue compatibility and infection elimination were confirmed by histological investigation [49].

Collectively, these findings show that curcumin-based nanoplatforms can safely and effectively eliminate harmful bacteria and biofilms, promote wound healing, and provide strong protection against antibiotic resistance when activated by light (and occasionally ultrasound). The variety of curcumin formulations highlights the versatility and therapeutic promise of curcumin as a photosensitizer in sophisticated aPDT protocols.

### 3.2. Perylenequinones

Generally, perylenequinones are naturally occurring pigmented secondary metabolites primarily found in fungi, but also in lichens, bacteria, protozoa, and some marine organisms, with some rare exceptions in plants. Some of them can be extracted from the *Fagopyrum esculentum*, commonly known as herb buckwheat, aphids such as *Hormaphis* spp., and the protozoan *Blepharisma* spp. [50].

Perylenequinones are effective singlet oxygen producers; hence, a PDT type II mechanism is widely recognized, and they have low cellular toxicity. In particular, hypocrellin A (HA), isolated from *Hypocrella bambusae*, showed potential as a natural PS for antimicrobial therapy. In a recent study, the antibacterial effect of hypocrellin A photoactivated by 470 nm LED light was studied against *Cutibacterium acnes*, a Gram-positive bacterium usually associated with acne vulgaris. The increase of light dose (10 J/cm^2^, 30 J/cm^2^, 50 J/cm^2^) determined the decrease of MIC and MBC values, corresponding to 1 μg/mL, 0.125 μg/mL and 0.03125 μg/mL for MIC and 4 μg/mL, 1 μg/mL and 0.25 μg/mL for MBC. By means of transmission electron microscopy (TEM) and scanning electron microscopy (SEM) studies, disrupted cell membranes and cytoplasmic leakage in HA-PDT-treated bacteria, compared to intact control cells, have been shown. Finally, a mild cytotoxic effect on HaCaT keratinocytes was observed at 1 μg/mL, more pronounced when light was applied, but no significant toxicity was exhibited at concentrations below 0.5 μg/mL. These results offered further evidence of HA’s potential for safe use in clinical settings to treat acne [51].

**Figure 5:**
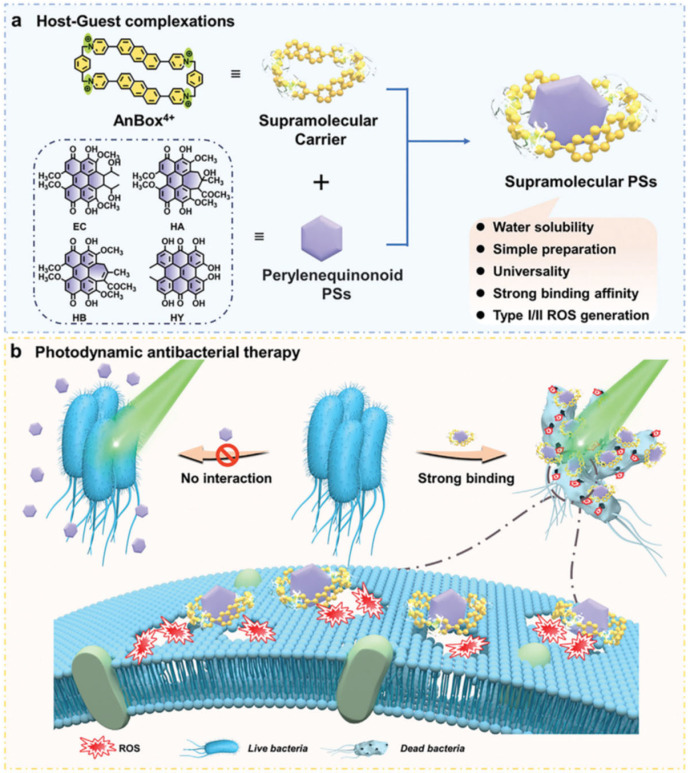
Scheme of (**a**) structure of water-soluble cationic macrocyclic carrier (AnBox·4Cl) complexed with perylenequinonoid PSs and (**b**) antimicrobial photodynamic effect on bacteria. Reprinted from [53] under Creative Commons License. Copyright © 2024 Wiley-VCH GmbH.

It has been demonstrated that perylenequinones are effective against Gram-positive bacteria but typically fail against Gram-negative bacteria due to poor membrane permeability and low water solubility [52]. To overcome this, Gao et al. employed a water-soluble cationic macrocyclic carrier (AnBox·4Cl) that forms complexes with perylenequinonoid photosensitizers (including elsinochrome C, hypocrellin A, hypocrellin B, and hypericin), significantly boosting their antibacterial efficacy and encouraging the membrane anchoring capability. After 532 nm laser irradiation (100 mW/cm^2^), both tested Gram-positive (*S. aureus*) and Gram-negative bacteria (*E. coli* and *P. aeruginosa*) reported a significant photodynamic antibacterial effect with bactericidal rates over 90% at a concentration of 2.5 μM (Figure 5) [53].

#### 3.2.1. Hypericin

Among natural sources of perylenequinones, *Hypericum perforatum*, commonly known as St. John’s Wort, is a flowering plant traditionally recognized for its healing properties, particularly in treating burns and skin injuries. Clinical studies have also demonstrated that this plant possesses antiviral, antidepressant, antibacterial, and antitumor properties. One of the key active compounds in *H. perforatum* is hypericin, which exhibits optimal absorption at a wavelength of approximately 550–600 nm, corresponding to orange-colored light. In recent decades, hypericin has been intensively studied for its broad pharmacological spectrum. Among its antidepressant, antineoplastic, antitumor, and antiviral activities, hypericin functions as a potent natural photosensitizer, demonstrating potential utility in antimicrobial photodynamic therapy [54]. For ophthalmologic applications, for instance, it has been demonstrated that hypericin can bind to the lens protein systems, not impairing their chaperone-like activities and thus might act as a potential photoagent against ocular infections [55]. Photoactivated hypericin proved to be largely effective against Gram-positive bacteria, such as *E. faecalis*, *P. acnes*, and *S. aureus* (even MRSA). In detail, 75 ng/mL hypericin irradiated with orange LEDs (590 ± 10 nm, 6 J/cm^2^) was able to promote the bacterial death of *E. faecalis* up to 99%, without affecting host tissue. However, 7500 ng/mL hypericin and a higher dose of light (12 J/cm^2^) were needed to reduce the bacterial growth to 58% in biofilms [56]. Even in the case of *S. aureus* and MRSA, 10 μM of hypericin irradiated with orange light (100 W/m^2^) for 1 h was able to reduce the CFU/mL value of ~6 log_10_, compared to the ampicillin-treated control [57]. Instead, the treatment with hypericin and light of *P. acnes* biofilm underlined the importance of the appropriate selection of molecule concentration and light dose, proving that the combination of the highest tested dose light (166 J/cm^2^) with the highest concentration of hypericin (15 μg/mL) induced a significant biofilm reduction of 27.9%, compared to the untreated control [58]. On the other hand, Gram-negative bacteria are more resistant to photoactivated hypericin, probably due to their additional outer membrane and permeability barrier consisting of lipopolysaccharides. An example is the treatment of *P. aeruginosa* with 10 μM hypericin activated with orange light (100 W/m^2^) for 3 h, which did not show any killing effect [57]. Although hypericin has many desirable properties, including a high quantum yield of singlet oxygen generation, low dark toxicity, a high extinction coefficient close to 600 nm, and a notable inhibition of gram-positive bacterial growth, its high lipophilicity and water insolubility in its natural form limit its use in biological applications [59].

#### 3.2.2. Hypericin in Combination with Nanostructures

To overcome the main disadvantages of hypericin related to its clinical implementation, scientists focused on the synthesis of nanoparticles and liposomes mimicking the lipidic cellular membrane microenvironment as carriers of the PS [60].

**Figure 6:**
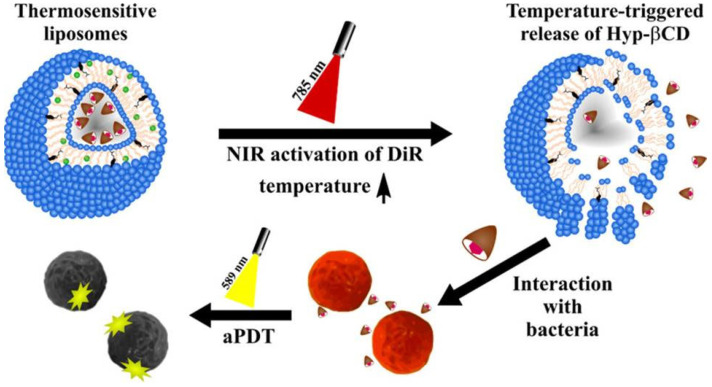
Schematic illustration of photothermal-triggered release and synergistic photodynamic therapy against *Staphylococcus saprophyticus* of near-infrared activated thermosensitive liposomes loaded with the near-infrared (NIR)-dye 1,1-dioctadecyl-3,3,3,3-tetramethylindotricarbocyanine iodide (DiR) and the water-soluble hypericin (Hyp) β-cyclodextrin inclusion complex (Hyp-βCD). Figure reprinted from [62] under Creative Commons Attribution 4.0 International License.

Hypericin (HYP)-based formulations of aPDT have shown remarkable success in combating biofilm-forming and multidrug-resistant bacteria. Malacrida et al. produced Pluronic^®^ P123 polymeric nanoparticles with two different [hypericin]/[P123] molar ratios (2.5 and 10, containing 50 μmol/L and 100 μmol/L, respectively) and tested them against Staphylococcus aureus. The bacterial photoinactivation (using orange LED light, 0.54 J/cm^2^) was observed in concentrations of 12.5 to 3.12 μmol/L for HYP/P123-2.5 and reductions of ~4.0 log CFU/mL in 12.5 to 0.78 μmol/L for HYP/P123-10. No antibiofilm activity was observed for either formulation after 30 min of irradiation [61]. Besides, the assembly of thermoresponsive liposomes co-loaded with hypericin-β-cyclodextrin complexes and photothermal dyes demonstrated the effectiveness of liposomal delivery methods, reducing *Staphylococcus saprophyticus* by more than 4 log_10_ after near-infrared activation (Figure 6) [62].

APDT was also combined with antimicrobial sonodynamic therapy (aSDT) to enhance the antimicrobial effect. In particular, when activated by light or ultrasound, hypericin nanoparticles (HypNPs), which are frequently mixed with co-agents like D-tryptophan (D-Trp) or antimicrobial peptides, have demonstrated strong synergistic activity, improving bacterial inactivation and biofilm disintegration. For example, under photo-sonodynamic circumstances, HypNPs and D-Trp, which have a potent membrane-disruptive action, reduced the Gram-negative *A. baumannii* viability by 5.1 log_10_ CFU/mL and downregulated the expression of virulence genes (including *abaI*) by more than ten times [63]. These investigations highlight the therapeutic potential of customized aPDT formulations that target both planktonic and biofilm-embedded bacteria by employing hypericin derivatives.

### 3.3. Tetrapyrrolic Macrocycles

Tetrapyrrolic macrocycles include natural compounds like cobalamin, heme, and chlorophyll, which are referred to as the “pigments of life” because of their abundance in nature. The most often utilized PSs for aPDT investigations are porphyrins and hydroporphyrins.

Porphyrins and their derivatives consist of four pyrrole subunits joined by methine bridges and have outstanding photochemical, photophysical, and antimicrobial characteristics, such as high singlet oxygen yields, high ROS output, photostability, effective absorption of visible light, and low dark toxicity. Porphyrins have a strong absorption in the 410–420 band (Soret band) and a non-intense absorption band in the region of 500–700 nm [64]. On the other hand, unsaturated hydroporphyrins, which include bacteriochlorins and chlorins, are abundant in nature and have significant biological functions. They belong to the second generation of PSs and are distinguished by their strong absorbance in the 600–750 nm range, reduced stability, and susceptibility to oxidation into porphyrins when exposed to ambient oxygen [65].

#### 3.3.1. Endogenous Porphyrins

**Figure 7:**
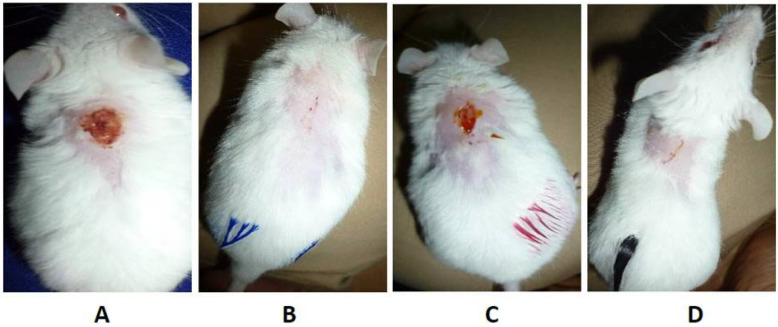
Irradiation treatment in murine wound models consisting of (**A**) no irradiation, (**B**) irradiation with LED 391.76 nm, (**C**) irradiation with LED 627.73 nm, and (**D**) irradiation with the combination of 391.76 nm and 627.73 nm. Reprinted from [70] under Creative Commons Attribution 3.0 License.

It is well known that some bacterial species can produce endogenously different kinds of porphyrins in different amounts. In particular, porphyrins are intermediate species in the heme biosynthesis, which starts from a charged glutamyl-tRNA and passes through the intermediate species aminolevulinic acid (ALA) until the creation of coproporphyrinogen. At this stage, coproporphyrinogen III can be oxidized to coproporphyrins (I and III) or decarboxylated to protoporphyrinogen IX and subsequently oxidized to protoporphyrin IX [66]. They feature a high absorption peak around 400 nm (Soret band) and four smaller bands in the 500–650 nm range, allowing them to be activated by blue, green and red light as well [67]. Studies conducted in vitro and in vivo on a variety of Gram-positive and Gram-negative bacterial species exploited these bacterial PSs for aPDT. It was demonstrated that Gram-positive bacteria, such as *S. aureus*, when exposed to blue light at dosages of 100–150 J/cm^2^, exhibit great sensitivity, leading to bacterial reductions surpassing 3 log_10_ in CFU/mL [68,69]. The antimicrobial function of photoactivated endogenous porphyrins was also confirmed by the reduced efficiency in ∆hemB mutants, which are lacking in heme production [69]. Some researchers also applied a dual-wavelength strategy in order to enhance the antimicrobial effect. Using 392 nm (blue) and 628 nm (red) LEDs, Astuti et al. showed that aPDT produced the most effective inactivation of *S. aureus* (up to 67.1%) at energy densities of about 19.44 J/cm^2^ [70]. Moreover, Leanse et al. first sensitized the bacteria with 460 nm light, photolyzing staphyloxanthin, a carotenoid that scavenges ROS, and subsequently exposed them to 405 nm light. This two-step method increased bacterial death in vitro from about 1 log_10_ to more than 3 log_10_ reduction in CFU/mL when compared to 405 nm alone [68]. The efficacy of this strategy was also demonstrated in vivo in murine wound models infected with S. aureus, proving a strong bacterial inactivation and the best rate of wound healing with no evidence of tissue apoptosis (Figure 7) [68,70]. Similarly, latent cells of *Mycobacterium smegmatis*, previously pre-conditioned with zinc and magnesium to raise porphyrin levels, demonstrated greater susceptibility to 565 nm light at 180 mW/cm^2^ for 30 min [71].

**Figure 8:**
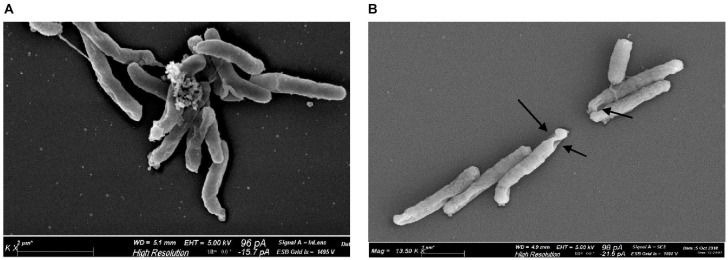
Scanning electron microscope imaging of *H. pylori* cell morphology before and after irradiation at 405 nm. Arrows indicate the presence of holes in the cell wall of irradiated bacteria. Reprinted from [74] under the terms of the Creative Commons Attribution 4.0 License.

In contrast, although they also produce porphyrins, Gram-negative bacteria like *Helicobacter pylori*, *Pseudomonas aeruginosa*, *Neisseria gonorrhoeae*, *Aggregatibacter actinomycetemcomitans*, and *Porphyromonas gingivalis* typically need higher doses for comparable killing because of their outer membrane barriers, which restrict light penetration and porphyrin accessibility. While *N. gonorrhoeae* obtained a 3.12 log_10_ decrease in CFU/mL and the complete bacterial eradication at 405 nm with dosages of 27 and 54 J/cm^2^ (60 mW/cm^2^), respectively, *P. aeruginosa* demonstrated up to 3 log_10_ CFU/mL reduction with 411 nm light at 15.7 mW/cm^2^ and doses up to 150 J/cm^2^ [72,73]. Accordingly, using 405 nm light at doses of 9–22 J/cm^2^ and irradiances of 21–41 mW/cm^2^, depending on the strain, *H. pylori* demonstrated morphological surface damage consistent with ROS-mediated death and up to 5 log_10_ CFU/mL decrease (Figure 8) [74]. Depending on the growing medium, *A. actinomycetemcomitans* needed a more intense exposure (403 ± 15 nm at 588 mW/cm^2^ for 30 min) to reduce CFU by 96% to >99.9% [75]. Moreover, a dosage of 30 J/cm^2^ of 405 nm light significantly reduced *P. gingivalis* while leaving host fibroblasts unaffected. Light-induced damage was facilitated by transcriptomic analysis, which revealed downregulation of ROS-scavenging pathways and overexpression of heme uptake and iron export genes [76]. In vivo studies validated the antimicrobial effectiveness of photoactivated endogenous porphyrins in Gram-negative bacteria. Bioluminescent *P. aeruginosa* infection in a mouse skin abrasion model was eliminated with a single 415 nm light exposure (48 J/cm^2^ at 100 mW/cm^2^), resulting in an average 5 log_10_ decrease in bacterial luminescence and, even after ten sublethal exposure cycles, no bacterial tolerance was developed [77]. Furthermore, in another study, *P. aeruginosa* virulence was investigated using a *Caenorhabditis elegans* infection model. Worm lifespan dramatically increased after the irradiation with blue light (10 J/cm^2^ at 15.7 mW/cm^2^), while bacterial counts remained unchanged, suggesting that aPDT decreased pathogen pathogenicity and biofilm formation instead of killing the bacteria directly [73].

#### 3.3.2. Exogenous Porphyrin Precursor and Porphyrins

The committed precursor 5-aminolevulinic acid (ALA) could represent a limit for the rate of heme biosynthesis, decreasing the formation of endogenous porphyrins. For this reason, different studies demonstrated that adding ALA significantly increases the production of coproporphyrin III (CPIII) and protoporphyrin IX (PPIX) in a variety of bacterial species, including *Klebsiella pneumoniae*, *E. coli*, *Corynebacterium diphtheriae*, *S. aureus*, and *Mycobacterium abscessus* [78,79,80,81]. In particular, when exposed to ALA, CPIII increased strain-dependently in *C. diphtheriae*, which was directly correlated with improved photoinactivation efficiency on both planktonic cells and biofilm-embedded bacteria and shorter light exposure durations [80]. In addition, the capacity of ALA to augment porphyrin-mediated ROS production facilitated the effective eradication of biofilms in polymicrobial wound infections, also constituted by bacterial species usually unable to produce high endogenous porphyrin levels [78]. In vivo studies further highlight the therapeutic benefits of exogenous ALA. When applied topically to MRSA-infected mouse wound models, ALA significantly increased PPIX accumulation and promoted both fluorescence-guided diagnosis and red light-mediated bacterial inactivation, leading to a decrease in viable bacterial counts [82]. With strong effectiveness against planktonic and biofilm-embedded bacteria in vitro and in vivo, research jointly highlights the critical function of ALA in promoting porphyrin production for therapeutic interventions as well as diagnostic imaging in photodynamic antimicrobial methods.

Recent research has shown that exogenous porphyrins, especially protoporphyrin IX and coproporphyrin III, can also be used as antimicrobial agents to facilitate the photodynamic inactivation of harmful bacteria that are not able to produce endogenous photosensitizers. For example, even though *Streptococcus agalactiae* produces the blue light-absorbing chromophore granadaene, it is resistant to 450 nm blue light because it is not able to generate ROS. Despite this, either PPIX or CPIII supplementation allowed for a notable reduction in bacteria after irradiation, with CPIII outperforming PPIX at comparable quantities. *S. agalactiae* most likely internalized the porphyrins during incubation, which promoted the production of ROS and, when exposed to light, cell death [83]. In another study, Anane et al. used PPIX to study the photodynamic inactivation of multidrug-resistant *A. baumannii* biofilms. Their results demonstrated that when triggered by 652 nm light, PPIX caused a significant 6 log_10_ decrease in biofilm cell viability, despite its anionic nature and mild dark toxicity [84]. These recent results suggested that exogenous porphyrin-based photosensitization may be a potent weapon against planktonic and biofilm-associated bacterial infections, especially in strains that are resistant to antibiotics.

**Figure 9:**
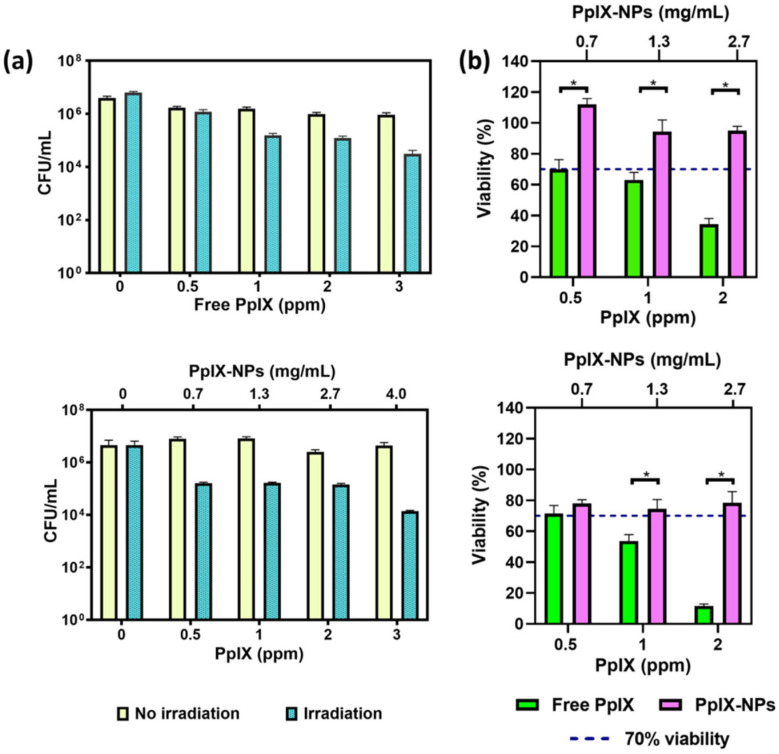
(**a**) Antibacterial tests against *S. aureus* employing free PPIX and PPIX-loaded nanoparticles; (**b**) Viability evaluations of PPIX released from PPIX-loaded NPs and free PPIX on fibroblasts after 1 and 24 h of incubation. * indicates significant differences between marked groups (*p* ≤ 0.001). Reprinted from [86] under Creative Commons Attribution 4.0 License.

Nevertheless, natural porphyrins are highly hydrophobic and can degrade quickly or aggregate in water, quenching excited states and ROS generation and reducing their photodynamic efficiency. Moreover, they can accumulate in healthy tissues and do not distribute specifically to the infection site, increasing the risk of side effects to host cells. For these reasons, nowadays researchers are increasingly exploiting synthetic porphyrins, such as cationic porphyrins, or nanotechnology-based strategies to obtain more efficient, precise, and safer antimicrobial effects of photodynamic therapy [65,85]. Focusing on the optimization of delivery to enhance photodynamic efficacy, PPIX encapsulated in PLGA nanoparticles showed preserved photodynamic activity against *S. aureus*, improved photostability, and reduced cytotoxicity in human fibroblasts (Figure 9). Under 532 nm illumination at 0.5 W/cm^2^ for 5 min, the nanoparticles provided regulated release and achieved 2 log bacterial decrease at dosages ranging from 0.5 to 10 ppm [86]. Another method was using NewPS, a porphyrin-shelled nanoemulsion devoid of surfactants that allowed for efficient delivery across biofilms and in models of infected tissue. When combined with 660 nm light at 60 J/cm^2^, NewPS induced up to 6 log reductions in CFU/mL of *S. aureus* and *Streptococcus pneumoniae*, utilizing as little as 0.5 nM in planktonic forms and 100 μM in biofilm conditions. Significantly, longer drug-light intervals increased biofilm penetration and treatment effectiveness, indicating that formulation stability and incubation duration are important factors that affect the effectiveness of therapy [87]. Besides, by conjugating PPIX to bismuth ferrite (BFO) harmonic nanoparticles, Kumar et al. enabled the second harmonic generation (SHG)-mediated activation under near-infrared (NIR) femtosecond pulsed laser irradiation, transforming 798 nm NIR photons into 399 nm light. This strategy permitted the overcoming of a basic drawback of traditional porphyrins: their poor activation under tissue-penetrating NIR light. In contrast to the negligible effects of PPIX (76.7%) or BFO (84.0%) alone, the BFO-PPIX conjugates demonstrated considerably increased photodynamic effectiveness against *S. aureus*, lowering bacterial survival to 44.5 ± 3.4% after 8 min of laser irradiation [88]. The eradication of *S. aureus* and *E. coli* was also observed after the light irradiation (532 nm) for 10 min of a multifunctional nanoplatform composed of zinc oxide nanoparticles as a delivery mechanism, berberine as an antimicrobial agent, porphyrin as photosensitizer and sodium alginate natural polymer. The nanoformulations showed moderate biocompatibility on normal human immortalized retinal epithelial (RPE1) cells, according to the findings of the cytotoxicity evaluation [89]. Even though porphyrin-based nanomaterials’ aPDT shows distinct benefits in terms of antibacterial and antibiofilm properties, it is still in its early stages and has several obstacles to overcome due to complex synthesis procedures and high production cost [90].

### 3.4. Flavins

Flavins are a family of yellow-colored heterocyclic chemical compounds found in biological systems. Riboflavin, commonly known as vitamin B2, is an essential component of living organisms and is the precursor of all biologically important flavins such as flavin mononucleotide (FMN) and flavin adenine dinucleotide (FAD) [91]. They are generated from riboflavin (vitamin B2), which is also the prevalent flavin applied in aPDT. These cofactors are essential for redox processes, including oxidative metabolism and the electron transport chain. Flavins can act as endogenous photosensitizers because of their double-bonded cyclic structures, which can significantly absorb in the blue portion of the visible spectrum (440–470 nm) and in the ultraviolet A (UVA) region (360 nm).

#### 3.4.1. Endogenous Riboflavins

**Figure 10:**
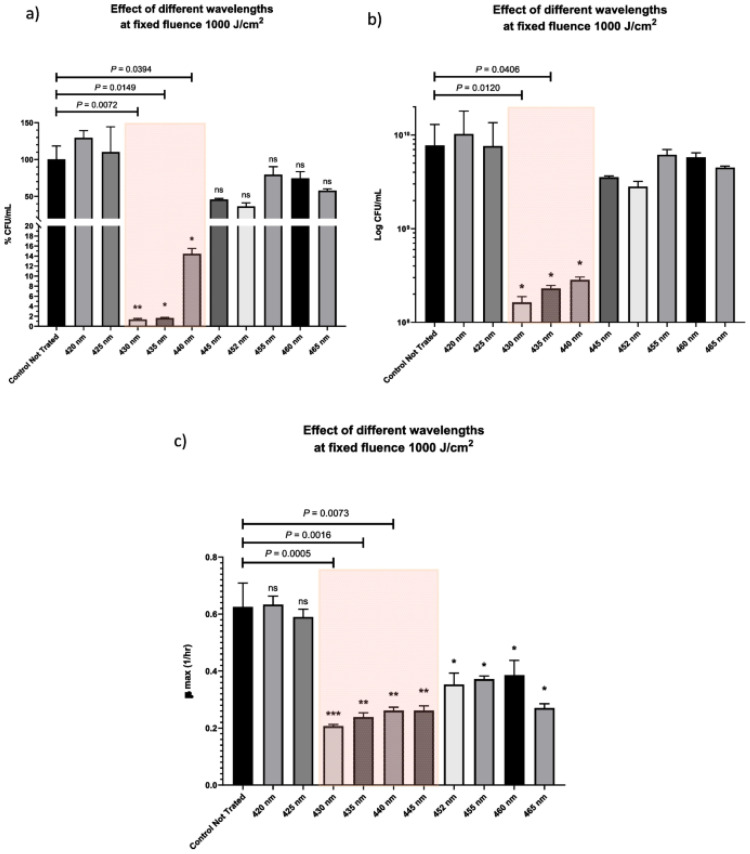
In terms of (**a**) percentage CFU/mL, (**b**) log CFU/mL, and (**c**) growth rate (μmax) during the logarithmic phase of growth, the bar graphs compare the control culture with bacterial cultures that were exposed to various laser parameters at ten distinct wavelengths at a fixed fluence of 1000 J/cm^2^. ANOVA and Tukey test determined the statistical significance: *** highest significance (*p* < 0.0001), ** highly significant (*p* < 0.001), * low significance (*p* < 0.05), and “ns” non statistically significant. Figure reprinted from [92] under Creative Commons Attribution 4.0 International License.

The vitamin riboflavin, which is also present in bacterial cells, is often crucial for their sensitivity to light conditions. Its use in antimicrobial photoinactivation is made possible by its photoreactivity; thanks to the flavin-induced oxidative stress, microbial cells can be destroyed without the need for external photosensitizers [17,66]. For example, femtosecond laser irradiation at 430–445 nm resulted in up to 98.6% bacterial inactivation in *E. faecalis*, which lacks endogenous porphyrins. This is compatible with the absorption spectra of flavins and validates their function as principal photosensitizers (Figure 10) [92]. Similarly, transcriptome evidence of oxidative stress responses and in vitro ROS detection experiments demonstrated that high-power blue laser illumination at 448 nm quickly inactivated *Escherichia coli* by causing ROS-mediated damage, especially through flavin excitation [93]. Porphyrins and flavins were both found in *N. gonorrhoeae*, and although the photoactivation at 405 nm was more effective than at 470 nm, the latter still had a strong phototoxic effect, demonstrating the role of flavins in photoinactivation [72]. Moreover, in addition to the previously known porphyrin-mediated processes, fluorescence spectroscopy and mass spectrometry in *H. pylori* revealed flavin chemicals, particularly riboflavins, as important contributors to bacterial death under irradiation at 460 nm [74].

#### 3.4.2. Exogenous Riboflavins

Because of its water solubility and safety, riboflavin was also used as an exogenous photosensitizer in order to treat different infections caused by both Gram-positive and Gram-negative bacteria. Numerous in vitro studies proved that photoactivated riboflavin has strong inhibitory effects on *S. mutans* in both its planktonic and biofilm phases. Riboflavin-mediated aPDT dramatically decreased *S. mutans* CFU counts and biofilm mass, and higher laser power densities (up to 1.0 W/cm^2^) produced stronger bactericidal effects. Crucially, the oxidative processes triggered by PDT helped to partially overcome the biofilm-specific difficulties, including extracellular polymeric substances protection and metabolic dormancy [94,95,96,97]. Its efficacy in reducing bacterial adhesion and biofilm formation was also shown in clinically relevant scenarios, such as the area surrounding orthodontic brackets and denture acrylics [98,99]. Furthermore, when riboflavin PDT was coupled with postbiotic mediators, Pourhajibagher et al. observed decreased virulence gene (*gtfB*) expression and increased biofilm disintegration [100]. The planktonic bacterial reduction was also proven for other species. For example, riboflavin irradiated by 450 nm diode laser at 400 mW for 120 s (fluence 48 J/cm^2^) induced up to 95.9% reduction in *S. aureus* [101], and at a dosage of 0.2 kJ/cm^2^ at 460 nm, *Listeria monocytogenes* was reduced by 5.6 log_10_ CFU/mL in phosphate-buffered saline (PBS) at 100 μM riboflavin [102]. Similarly, irradiating at 440 nm with a LED light, *A. baumannii* planktonic cells were decreased by up to 2.92 log_10_ CFU at 158.4 J/cm^2^ [103]. Moreover, four antibiotic-resistant strains (MRSA, *A. baumannii*, extended-spectrum beta-lactamase-producing *E. coli*, and *K. pneumoniae*) were inactivated by more than 80% by photodynamic inactivation in whole blood using riboflavin and ultraviolet light (308 and 365 nm) at 18 J/cm^2^. Higher doses increased efficacy further while preserving low hemolytic effects [104]. Although the protective matrix typically resulted in smaller decreases, riboflavin-mediated aPDT remained effective against biofilms. For instance, under comparable irradiation conditions, riboflavin decreased *A. baumannii* biofilms more than chlorophyllin [105], while *E. faecalis* biofilms treated with riboflavin with a 450–460 nm laser or LED (12–60 J/cm^2^) experienced a significant decrease in biofilm biomass and structural disruption [106,107].

The antimicrobial effect of riboflavin was also enhanced by the synergistic use of antibiotics. Mills et al. created a riboflavin-vancomycin compound (VanB2) that completely photodynamically eliminated vancomycin-resistant *E. faecalis* biofilms, resulting in a more than 7 Log_10_ decrease in bacterial viability. This method greatly increased biofilm penetration and killing effectiveness by combining two modes of action: light-triggered antibiotic release and localized singlet oxygen generation as well [108]. Furthermore, when combined with riboflavin and 460 nm LED irradiation (60 J/cm^2^), dosages of colistin below the minimum inhibitory concentration reduced *P. aeruginosa* viability, and biofilms were broken down by up to 5 logs [109]. The combined approach, which enhanced membrane permeability and improved phototoxicity, validated the promise of aPDT-antibiotic synergism in overcoming antimicrobial resistance.

**Figure 11:**
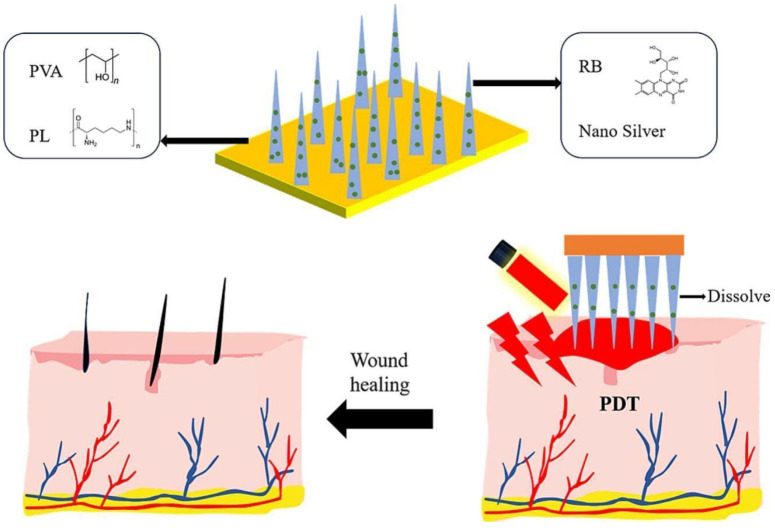
Schematic illustration of photodynamic bacterial biofilm removal using riboflavin-dispersing microneedles. Reprinted from [114], copyright © 2025, with permission from Elsevier B.V.

An additional strategy to improve the treatment of microbial infections with a precise delivery, localization, and release was the application of nanostructure-conjugated riboflavin systems, such as nanoparticles, chitosan hydrogels, carbon-polymerized dots, nanospheres, and dissolving microneedles. Interestingly, the synthesis of gold nanoparticles functionalized with riboflavin (Rf-AuNPs) showed a dose-dependent antimicrobial effect against *S. aureus* and *P. aeruginosa* when exposed to light at 365 nm, evidencing a 90% decrease in viable cells at concentrations as low as 50 μM of riboflavin. This increased effectiveness was ascribed to the localized surface plasmon resonance of AuNPs, which improved the energy transfer [110]. On the other hand, riboflavin-based carbon polymerized dots (Rf-CDs) demonstrated strong ROS production at 450 nm light (20 mW/cm^2^) and wide absorption in the visible range. Because of their enhanced absorption and photostability, the Rf-CDs outperformed free riboflavin, achieving a 5 log_10_ decrease in *E. coli* within 10 min of irradiation. The amphiphilic nature and nanoscale size allowed for deep penetration into bacterial membranes and biofilms [111]. Soleimani et al. showed that riboflavin-loaded chitosan hydrogels generated considerable singlet oxygen and antibacterial zones up to 18 mm in diameter against *S. aureus* when subjected to 450 nm LED light at 110 J/cm^2^ [112]. Similarly, Almoammar et al. added riboflavin-doped hydroxyapatite nanospheres to dental adhesives and activated them using UVA radiation at 375 nm (3 mW/cm^2^, 60 s), which significantly decreased the number of *S. mutans* and increased the strength of the microtensile bond [113]. Besides, riboflavin-loaded dissolving microneedles with 440 nm light were able to eradicate *S. aureus* and *E. coli* biofilms both in vitro and in vivo in a mouse subcutaneous abscess model with 96.3% drug release in 8 min and deep skin penetration (~384 μm) (Figure 11) [114]. By boosting photostability, ROS production, targeted distribution, and biofilm penetration, nanostructures provide a tactical edge in combating antibiotic resistance and enhancing localized PDT efficacy.

## 4. Conclusions and Future Perspectives

The emergence of antimicrobial resistance is increasingly becoming a global health concern that requires alternative therapeutic approaches beyond traditional antibiotics. Antimicrobial photodynamic therapy represents a potent alternative strategy, exploiting the generation of reactive oxygen species capable of fighting antibiotic-resistant bacteria both in planktonic and biofilm forms. Among the broad variety of photosensitizers, natural compounds, such as curcuminoids, perylenequinones (e.g., hypericin), tetrapyrrolic macrocycles (e.g., porphyrins), and flavins (e.g., riboflavin) have shown significant promise due to their low cytotoxicity, environmental sustainability, and biocompatibility.

For millennia, natural molecules have inspired chemists and clinicians. Their vast structural variety and complexity have motivated synthetic chemists to reproduce them in the lab, typically with therapeutic uses in mind, and “many drugs used today are natural products or natural-product derivatives” [115]. Natural compounds, thanks to their intrinsic biocompatibility and physicochemical characteristics, may really interact with and integrate into the molecular structure of biological systems. As a result, they can affect and, in many cases, disrupt both pathological and physiological processes. Nanotechnology advancements and innovative methods for natural-product isolation, characterization, and synthesis could open a new age of natural product research in academia and industry. In particular, despite their powerful antibacterial activity upon photoactivation, most of the natural photosensitizers exhibit poor solubility due to their hydrophobic nature, limited bioavailability, and suboptimal pharmacokinetics, which often restrict their clinical application. In this review, we have explored emerging drug delivery technologies that can be developed or optimized to address the limitations of natural photosensitizers in antibacterial photodynamic therapy. Nowadays, the majority of the research efforts are dedicated to exploring innovative delivery systems, including polymeric nanoparticles, liposomes, hydrogels and micelles. These nanostructures have demonstrated efficacy as drug delivery platforms for hydrophobic PSs, allowing for efficient transport both in vitro and in vivo. They facilitate the circumvention of physiological and biological obstacles, resulting in increased bacterial cell uptake. Specifically, polymeric (such as PLGA and chitosan), lipid and stimuli-responsive nanoparticles have been demonstrated to enhance PS solubility, preventing its self-aggregation, protect PS from degradation, facilitate tissue-specific targeting and long-term controlled release. Nanostructure formulations have recently advanced from simple micelles to multifunctional nanoplatforms capable of attacking and degrading both Gram-positive and Gram-negative multidrug-resistant bacteria, penetrating biofilms and tissues. The combination of natural PSs with photothermal agents such as plasmonic metals (Au, Cu, Pd, and Bi), carbon-based materials (carbon dots, GO, and graphene nanosheets), polymers (polydopamine, polyaniline, and polypyrrole), and semiconductors broadens the phototherapeutic window to NIR light (650–850 nm), which has a high penetration depth into human tissues and may increase antibacterial action due to the simultaneous formation of ROS and temperature rise. Furthermore, dual-mode therapies (aPDT + photothermal or sonodynamic) as well as the combination with antibiotics or postbiotics significantly enhance the clinical potential, particularly in dentistry, wound care, and systemic infection control.

Although it is commonly believed that antimicrobial photodynamic treatment (PDT) has little chance of causing bacterial resistance, there is some evidence that resistance may arise, especially at low doses of photosensitizers or through tolerance to reactive oxygen species (ROS). Thus, combining PDT with other therapies may be a promising strategy to effectively eradicate chronic infections.

As many bacterial infections involve biofilms or necrotic tissues with low oxygen, natural PSs with prevalent Type I mechanism, producing superoxide (O_2_^−^), hydroxyl radicals by means of electron transfer process without the need of molecular oxygen, could be advantageous. Many natural PSs can be synthetically modified to enhance Type I pathways (for example, conjugation with redox mediators), making them therapeutically suitable for hypoxic wounds, abscesses, or implant infections.

Future research should continue to focus on engineering bio-inspired photosensitizers, optimizing delivery systems, light sources and dosage. Designing and developing hybrid materials that include natural PSs with other nanomaterials, drugs, or photoagents can produce synergistic effects and will offer a comprehensive strategy for fighting microbial diseases. Ongoing research on scalable and economical production methods is essential to increase the accessibility of aPDT for medical applications.

According to previous studies, the safety profile and multifunctionality of natural photosensitizers, along with the adaptability of aPDT, make this approach a strong supplement and in many cases a powerful substitute for traditional antimicrobials.

## Figures and Tables

**Table 1:** Photophysical properties and therapeutic applications of examples of natural PSs.

PS	Absorption Bands (nm)	^3^O_2_ Quantum Yield (ΦΔ)	Solubility (in H_2_O)	Photostability	PDT Mechanism	Antimicrobial Mechanisms	Clinical Applications	Refs
**Curcumin**	420–430 nm	~0.36–0.50	Poor	Low (rapid degradation, light-sensitive)	Mainly Type I	Membrane and protein oxidation	Wound healing, oral infections, and dental caries	[17]
**Hypocrellin A**	465–470 nm	~0.85	Moderate	Moderate (sensitive to light and oxygen)	Type I and II	Membrane disruption and mitochondrial dysfunction	Skin infections	[18]
**Hypericin**	545–590 nm (visible), 590–610 nm (red edge)	~0.40–0.60	Very poor	High	Mainly Type II	Induces apoptosis via ROS only against gram positive bacteria and fungi.	Skin infections, biofilm-associated infections	[17,19]
**Porphyrin**	410–420 (Soret), 650–670 (Q-bands)	~0.65–0.80	Good to moderate	High	Mainly Type II	Membrane and DNA disruption and efflux pumps inactivation	Periodontic diseases, antibiotic-resistant infection	[19]
**Riboflavin**	440–460 nm	~0.55–0.60	Excellent	Moderate (photo-sensitive)	Type I and II	Biofilm disruption and the EPS matrix were damaged	Dental infections, sepsis models, and implant coatings	[16]

## Abbreviations

The following abbreviations are used in this manuscript:

aPDT	Antimicrobial Photodynamic Therapy
PS	Photosensitizer
AMR	Antimicrobial Resistance
WHO	World Health Organization
EPS	Extracellular Polymeric Substances
ROS	Reactive Oxygen Species
CUR	Curcumin
MRSA	Methicillin-Resistant *Staphylococcus aureus*
VRSA	Vancomycin-Resistant *Staphylococcus aureus*
CFU	Colony-Forming Unit
DHC	3,3′-dihydroxycurcumin
DAPMA	N,N-di-(3-aminopropyl)-N-methylamine
SPD	Spermidine
SPM	Spermine
NP	Nanoparticle
PLGA	Poly (lactic-co-glycolic acid)
MIC	Minimum Inhibitory Concentration
MBC	Minimum Bactericidal Concentration
PTT	Photothermal Therapy
AuNPs/CS	Chitosan-coated gold Nanoparticles
CD	Carbon Dot Composite
AC-Gel@	Hybrid hydrogel
AgTPP	Silver-Porphyrin Cyclodextrin and Phenylboronic acid
CurNisNP	Curcumin and Nisin in Nanoparticles
aPSDT	Antimicrobial Photo-Sonodynamic Treatment
GCDNH	Gold-Curcumin-Polydopamine Nanohybrid
HA	Hypocrellin A
TEM	Transmission Electron Microscope
SEM	Scansion Electron Microscope
AnBox·4Cl	Cationic Macrocyclic Carrier
HYP	Hypericin
aSDT	Antimicrobial Sonodynamic Therapy
D-Trp	D-Tryptophan
NIR	Near-Infrared
DiR	1,1-dioctadecyl-3,3,3,3-tetramethylindotricarbocyanine iodide
Hyp-βCD	Hypericin β-cyclodextrin
ALA	Aminolevulinic Acid
CPIII	Coproporphyrin III
PPIX	Protoporphyrin IX
BFO	Bismuth Ferrite
SHG	Second Harmonic Generation
RPE1	Human Immortalized Retinal Epithelial (cells)
FMN	Flavin Mononucleotide
FAD	Flavin Adenine Dinucleotide
UVA	Ultraviolet A
PBS	Phosphate-Buffered Saline
Rf-AuNP	Gold Nanoparticle functionalized with Riboflavin
Rf-CD	Riboflavin-based Carbon polymerized Dot
